# Recent advances in controllable/divergent synthesis

**DOI:** 10.3762/bjoc.21.73

**Published:** 2025-05-07

**Authors:** Jilei Cao, Leiyang Bai, Xuefeng Jiang

**Affiliations:** 1 Hainan Institute of East China Normal University, State Key Laboratory of Petroleum Molecular & Process Engineering, Shanghai Key Laboratory of Green Chemistry and Chemical Process, School of Chemistry and Molecular Engineering, East China Normal University, 3663 North Zhongshan Road, Shanghai 200062, PR China; 2 School of Chemistry and Chemical Engineering, Henan Normal University, Xinxiang, Henan 453007, PR China; 3 State Key Laboratory of Organometallic Chemistry, Shanghai Institute of Organic Chemistry, Chinese Academy of Sciences, 345 Lingling Road, Shanghai 200032, PR China

**Keywords:** controllable, divergent, diverse products, switchable synthesis

## Abstract

The development of streamlined methodologies for the expeditious assembly of structurally diverse organic architectures represents a paramount objective in contemporary synthetic chemistry, with far-reaching implications across pharmaceutical development, advanced materials innovation, and fundamental molecular science research. In recent years, controllable/divergent synthetic strategies for organic functional molecules using common starting materials have garnered significant attention due to their high efficiency. This review categorizes recent literatures focusing on key regulatory factors for product divergent formation, in which controlling chemical selectivity primarily relies on ligands, metal catalysts, solvents, time, temperature, acids/bases, and subtle modifications of substrates. To gain a deeper understanding of the mechanisms underlying reaction activity and selectivity differentiation, the review provides a systematic analysis of the mechanisms of critical steps through specific case studies. It is hoped that the controllable/divergent synthesis concept will spark the interest of practitioners and aficionados to delve deeper into the discipline and pursue novel advancements in the realm of chemical synthesis.

## Introduction

In the era of synthetic organic chemistry, divergence can produce stereodivergence (including diastereodivergence and enantiodivergence) [1–4] and regiodivergence [5–6]. In both cases, starting from the same substrate, different stereoisomers (diastereomers and enantiomers) or regioisomers can be obtained under different reaction conditions. Over the past two decades, researchers have found that by changing reaction conditions and modifying the substrate, two structurally distinct products that are neither stereoisomers nor regioisomers can be obtained from the same starting material (using the same reagents, if necessary), and significant progress has been made in recent years. Controllable/divergent synthetic strategies have increasingly attracted attention [5,7–14], for example, in 2024, Rana [15] and co-workers reported advances in solvent-controlled stereodivergent catalysis. Surprisingly, to our knowledge, there is currently no comprehensive review of studies on controllable/divergent synthesis. This review systematically examines, how these multidimensional control elements (including ligands, metal catalysts, solvents, time, temperature, acids/bases, and subtle modifications of substrates) synergize to achieve predictable product diversification. In addition, mechanistic insights are discussed providing illustrative examples across reaction classes, and emerging strategies for programming synthetic outcomes. The integration of these approaches promises to accelerate drug discovery and materials development through sustainable, atom-economic synthesis of complex molecular libraries.

## Review

### Ligand control

**Scheme 1:**
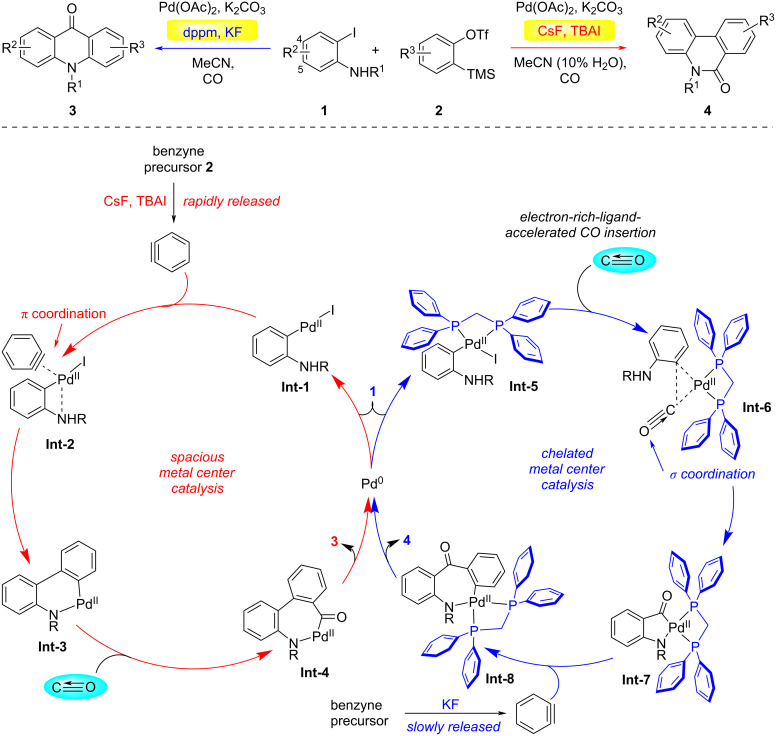
Ligand-controlled regiodivergent C1 insertion into arynes [19].

The precise regulation of product selectivity represents a fundamental challenge in transition-metal-catalyzed organic transformations, with significant implications for complex molecule synthesis. In this context, ligand-modulated divergent catalysis has emerged as a paradigm-shifting strategy, enabling programmable access to structurally distinct molecular architectures from identical substrate precursors through precise manipulation of metal coordination [16–18]. This sophisticated approach capitalizes on the stereoelectronic tunability of ancillary ligands to dictate reaction pathways, thereby offering unprecedented control over chemo-, regio-, and stereoselectivity parameters in catalytic manifolds. In 2015, the Jiang group developed a palladium-catalyzed regioselective three-component C1 insertion reaction (Scheme 1) [19]. In this reaction, an *o*-iodoaniline **1**, phenylacetylene, and carbon monoxide were used as starting materials, and two natural product frameworks of phenanthridone and acridone alkaloids could be selectively obtained by controlling ligands. The reaction of *o*-iodoaniline with in situ-generated arynes under CO atmosphere under ligand-free conditions selectively afforded phenanthridinones. Intriguingly, switching to the electron-rich bidentate ligand bis(diphenylphosphino)methane (dppm) redirected the pathway to yield acridones. Time-dependent NMR studies revealed that the selectivity hinges on the aryne release kinetics from its precursor. Employing CsF, tetrabutylammonium iodide (TBAI), and water significantly accelerated aryne generation, thereby increasing its local concentration. This favored aryne coordination to the palladium center, followed by CO insertion and reductive elimination to furnish phenanthridinones. In contrast, when dppm was introduced, oxidative addition of the C–I bond to palladium formed the four-membered aryl–palladium complex **Int-5**. Steric hindrance from the bulky dppm ligand, combined with slower aryne release (using KF as the fluoride source), attenuated aryne coordination. Under these electron-deficient conditions, CO preferentially occupied the palladium coordination site. Sequential insertion of CO and aryne, followed by reductive elimination, culminated in acridone formation. This ligand-dependent mechanistic dichotomy underscores the critical interplay between aryne availability, steric modulation, and electronic effects in steering catalytic selectivity.

**Scheme 2:**
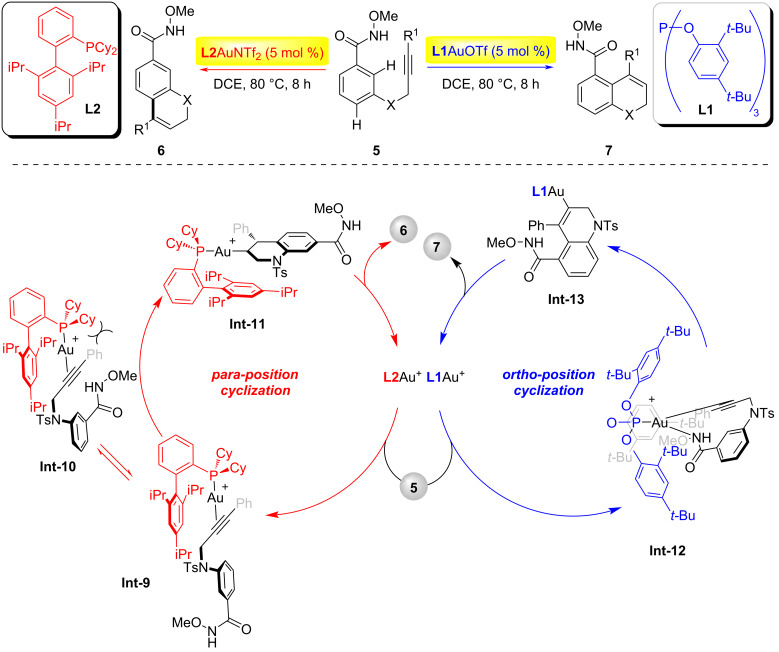
Ligand effect in homogenous gold catalysis enabling regiodivergent π-bond-activated cyclization [20].

In 2016, the Jiang group achieved regioselective control in the gold-catalyzed intramolecular hydroarylation of alkynes by modulating the electronic and steric effects of ligands (Scheme 2) [20]. Mechanistically, the electron-deficient phosphite ligand **L1** and the weakly coordinating OTf^−^ anion synergistically enhanced the electrophilicity of the gold center, enabling coordination with the amide group to form a three-coordinate Au(I)–π-alkyne intermediate **Int-12**. The umbrella-shaped steric shielding provided by the ligand-stabilized intermediate **Int-9**, followed by Friedel–Crafts-type addition and protonation to complete *ortho*-position cyclization. In contrast, *para*-position cyclization was exclusively achieved through π–π interactions between the electron-rich X-phos ligand and the substrate, compensating for the electron-deficient nature of the aromatic system and ensuring high regioselectivity.

**Scheme 3:**
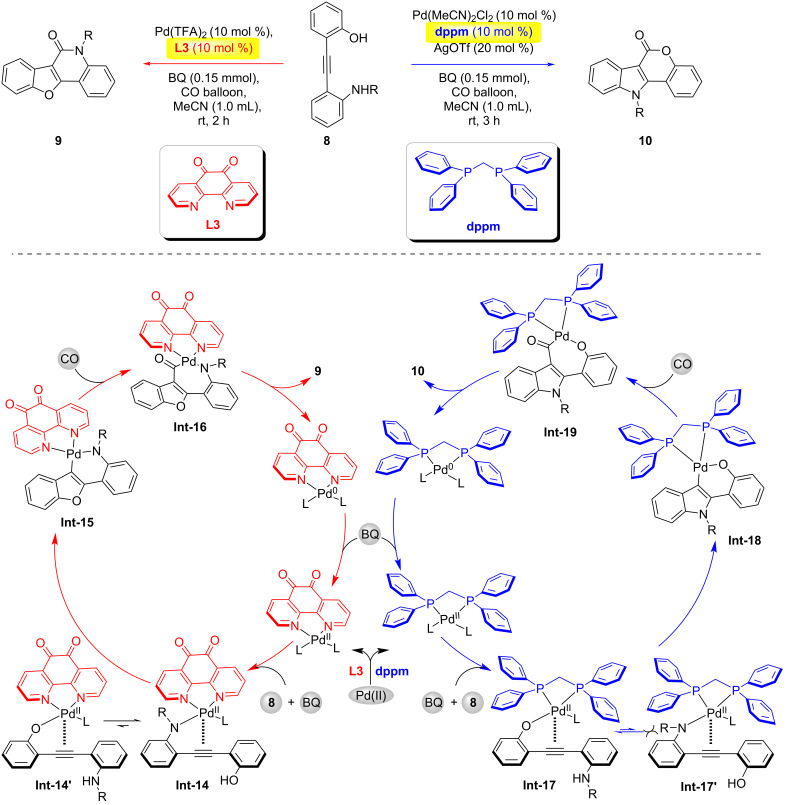
Ligand-controlled palladium(II)-catalyzed regiodivergent carbonylation of alkynes [21].

In 2018, the Jiang group developed a regiodivergent synthetic method for indolo[3,2-*c*]coumarins **10** and benzofuro[3,2-*c*]quinolinones **9** via controllable palladium(II)-catalyzed carbonylative cyclization (Scheme 3) [21]. When ligand **L3** coordinates with the palladium center, the enhanced electrophilicity of palladium facilitates preferential coordination with the amino group and activates the alkyne to form the intermediate **Int-14** instead of **Int-14'**. Subsequent nucleophilic cyclization generates intermediate **Int-15**. Following CO insertion, complex **Int-16** is formed, and reductive elimination yields the benzofuro[3,2-*c*]quinolinone product **9** along with a Pd(0) species, which is reoxidized to Pd(II) by BQ (benzoquinone). When the ligand is switched to the sterically bulky and electron-rich dppm, the chemoselectivity is reversed: the palladium center now preferentially coordinates with the hydroxy group to form complex **Int-17** and the amino group undergoes nucleophilic attack to generate **Int-18**. After CO insertion complex **Int-19** is produced and reductive elimination ultimately affords the indolo[3,2-*c*]coumarin product **10**.

**Scheme 4:**
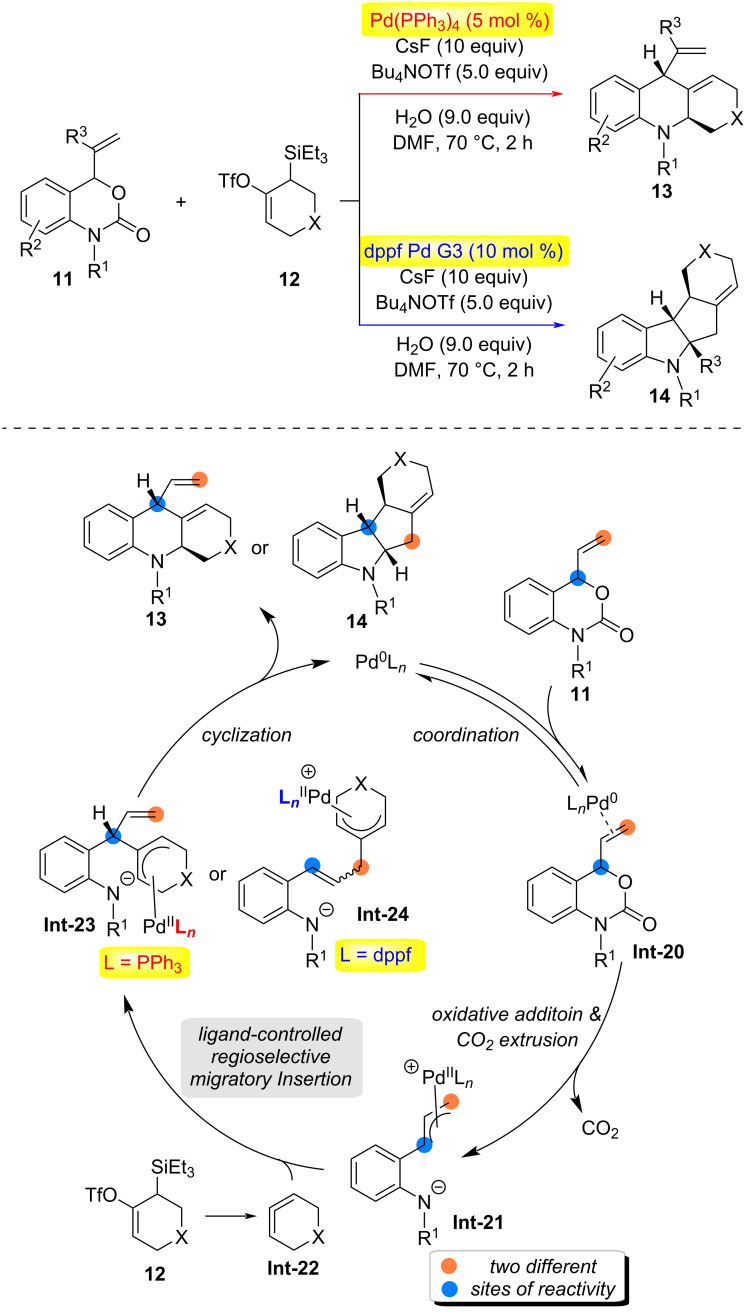
Catalyst-controlled annulations of strained cyclic allenes with π-allyl palladium complexes and proposed mechanism [22].

In 2023, the Garg group achieved the first example of utilizing in situ-generated π-allylpalladium complexes to capture strained cyclic allene intermediates (Scheme 4) [22]. By modulating the ligands in the reaction system, two distinct polycyclic scaffolds, **13** or **14**, could be synthesized with high selectivity. Mechanistically, the Pd(0) catalyst coordinates to substrate **11**, followed by oxidative addition and release of carbon dioxide to form the zwitterionic π-allylpalladium intermediate **Int-21**. Under the reaction conditions, silyl triflate **12** undergoes a fluoride-mediated 1,2-elimination to generate the cyclic allene intermediate **Int-22**. Through a ligand-controlled regioselective migratory insertion process, reaction of **Int-21** and **Int-22** leads to the formation of π-allylpalladium intermediates **Int-23** or **Int-24**, depending on the ligand employed. Finally, cyclization of **Int-23** or **Int-24** yields the tricyclic product **13** or the tetracyclic product **14**, respectively.

**Scheme 5:**
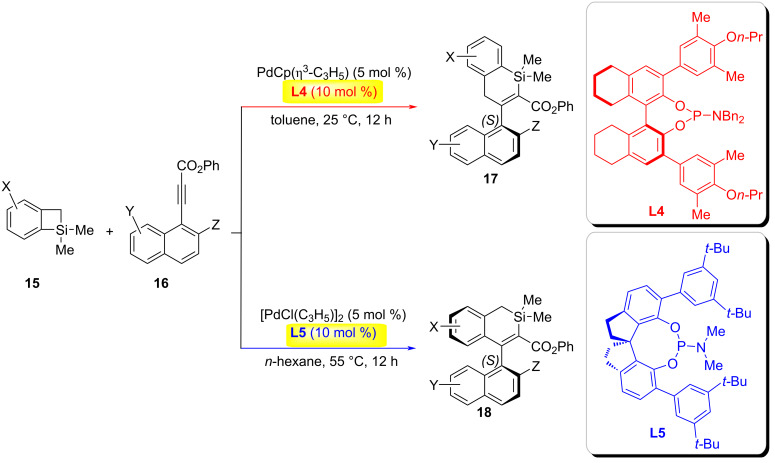
Ring expansion of benzosilacyclobutenes with alkynes [23].

In 2024, the Song group achieved a ligand-controlled regiodivergent and enantioselective ring expansion of benzosilacyclobutenes with internal naphthylalkynes by strategically modulating the ligand steric profiles (Scheme 5) [23]. Employing cavity-engineered phosphoramidite ligands, the reaction pathway bifurcated based on the steric demands of Si–C-bond activation. The methyl-substituted ligand (*S*)-8*H*-binaphthyl phosphoramidite **L4**, featuring a spacious cavity, favored sterically encumbered Si–C(sp^3^)-bond activation, selectively delivering axially chiral (*S*)-1-silacyclohexenyl arenes **17** with high enantiocontrol. Conversely, the bulky *tert*-butyl-decorated (*R*)-spirophosphoramidite **L5** imposed a confined cavity, steering selectivity toward Si–C(sp^2^)-bond activation and predominantly afforded the regioisomeric (*S*)-2-silacyclohexenylarenes **18**.

**Scheme 6:**
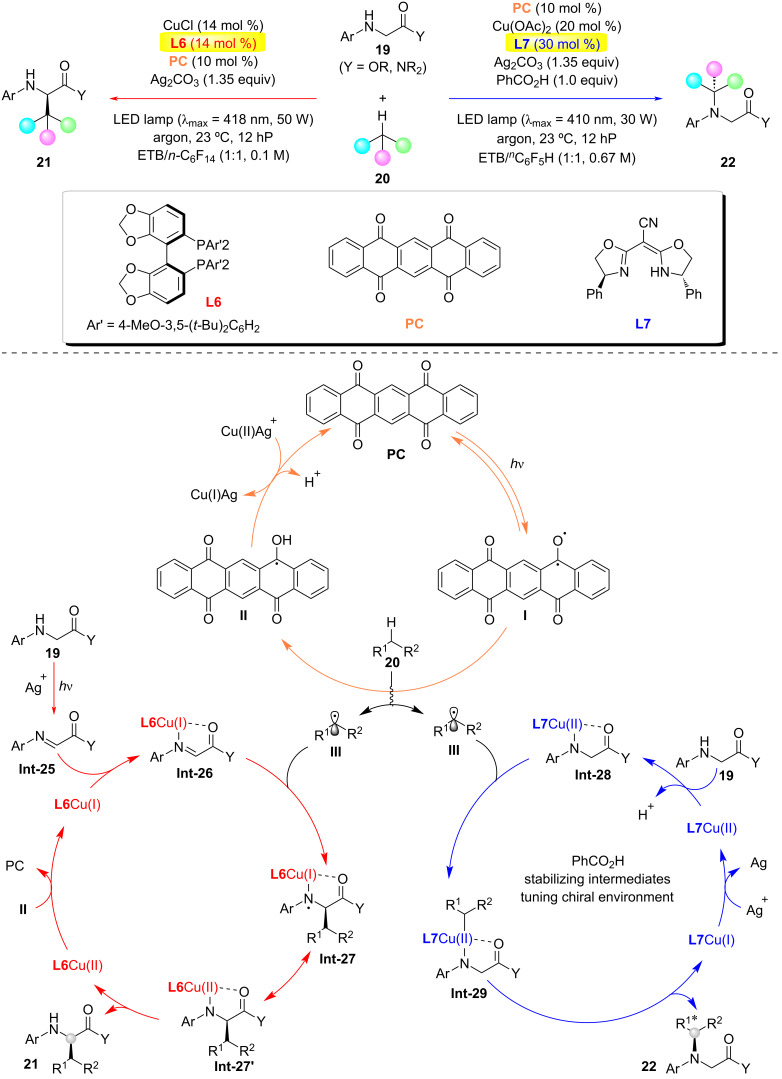
Photoinduced regiodivergent and enantioselective cross-coupling [24].

In 2025, Gong and co-workers reported a visible-light-mediated hydrogen atom transfer (HAT)/chiral copper dual catalytic system that achieved regiodivergent and enantioselective C(sp^3^)–C(sp^3^) and C(sp^3^)–N oxidative cross-couplings between *N*-arylglycine ester/amide derivatives and abundant hydrocarbon C(sp^3^)–H feedstocks (Scheme 6) [24]. This methodology also represents a highly challenging direct C(sp^3^)–H asymmetric amination. Mechanistic insights: When using a bulky, electron-rich chiral bisphosphine ligand **L6**, the glycine ester substrate coordinates with the copper catalyst to form a key intermediate complex **Int-26**. The sterically hindered and electron-rich environment around the copper center disfavors a direct interaction with nucleophilic alkyl radicals. Instead, the reaction proceeds via an outer-sphere mechanism, where the alkyl radical reacts with the copper-activated C=N unsaturated bond, enabling stereocontrolled C(sp^3^)–C(sp^3^) coupling. In contrast, with the anionic cyano-substituted bisoxazoline ligand **L7**, the glycine ester and copper catalyst form a distinct intermediate complex **Int-28**. The ligand’s reduced steric bulk and altered electronic properties facilitate direct interaction with alkyl radicals, forming a high-valent Cu(III) intermediate **Int-29**. This intermediate undergoes reductive elimination via an inner-sphere mechanism to generate the C(sp^3^)–N-coupled chiral product **22**. Notably, benzoic acid acts as a critical additive, likely by stabilizing key intermediates and modulating the steric/electronic environment for enhanced enantiocontrol.

### Metal control

**Scheme 7:**
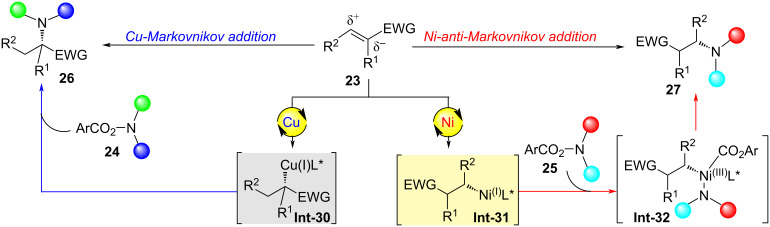
Catalyst-controlled regiodivergent and enantioselective formal hydroamination of *N*,*N*-disubstituted acrylamides [30].

Over the past decade, the relentless pursuit of precision in natural products and pharmaceutical synthesis has driven remarkable advances in catalytic methodologies, particularly in the realm of catalyst-controlled chemoselective transformations [11,25–29]. In 2023, the Shu group developed a catalyst-controlled regioselective and enantioselective hydroamination reaction of electron-deficient alkenes (Scheme 7) [30]. By efficiently regulating the regioselectivity and enantioselectivity of alkene **23** hydrometallation through catalytic systems, they overcame the influence of steric and electronic effects during the hydrometallation process, simultaneously achieving the synthesis of chiral α-quaternary carbon amino acid derivatives **26** and α-chiral β-amino acid derivatives **27**. Using a copper catalyst, the chiral α-quaternary carbon amino acid derivatives **26** were obtained with exclusive regioselectivity and excellent enantioselectivity. Employing a nickel catalyst, α-chiral β-amino acid derivatives **27** were synthesized with single regioselectivity and outstanding enantioselectivity.

**Scheme 8:**
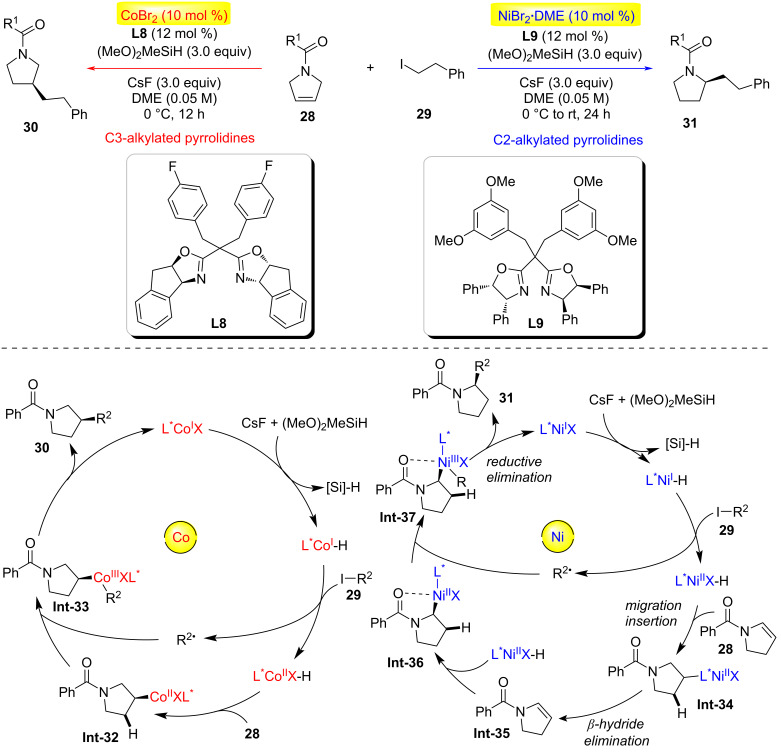
Catalyst-tuned regio- and enantioselective C(sp^3^)–C(sp^3^) coupling [31].

In the same year, Rong and co-workers reported a highly efficient catalyst-controlled regio- and enantioselective hydroalkylation reaction, enabling the divergent synthesis of chiral C2- and C3-alkylated pyrrolidines through desymmetrization of readily available 3-pyrrolines (Scheme 8) [31]. The cobalt catalytic system (CoBr₂ with modified bisoxazoline ligands) achieved asymmetric C(sp^3^)–C(sp^3^) coupling via distal stereocontrol, efficiently producing C3-alkylated pyrrolidines, while the nickel catalytic system afforded C2-alkylated pyrrolidines through a tandem alkene isomerization/hydroalkylation process. This method utilized readily accessible catalysts, chiral BOX ligands **L9**, and reagents, delivering enantioenriched 2-/3-alkyl-substituted pyrrolidines with excellent regio- and enantioselectivity (up to 97% enantiomeric excess). Radical-clock experiments and deuterium-labeled silane studies revealed that cobalt catalysis proceeded via irreversible Co–H migratory insertion to achieve C3 selectivity, whereas nickel catalysis involved alkene isomerization to generate a (2,3-dihydropyrrolyl) intermediate **Int-35**, followed by C2-selective coupling.

**Scheme 9:**
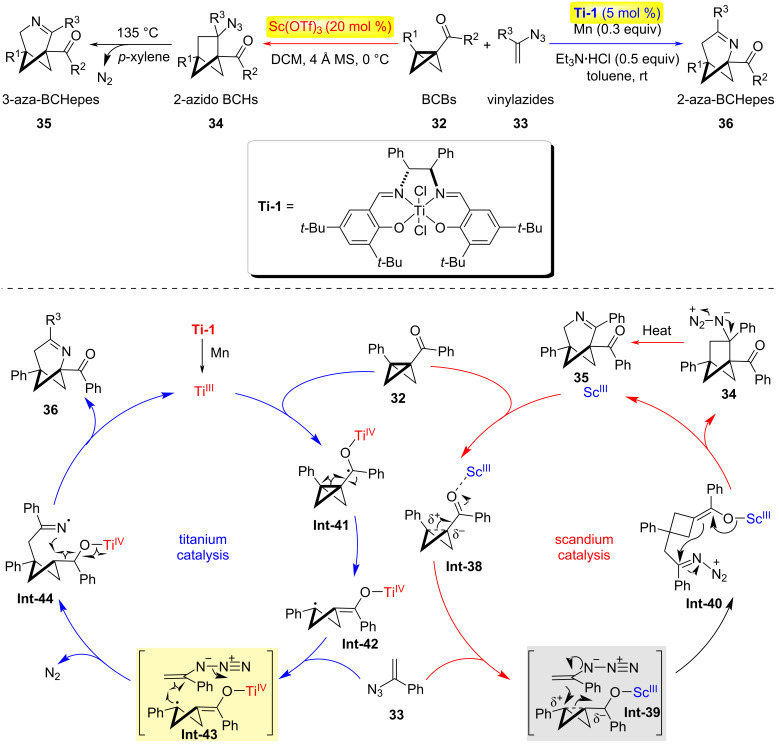
Catalyst-controlled annulations of bicyclo[1.1.0]butanes with vinyl azides [32].

In 2024, the Zheng group reported a catalyst-controlled cyclization reaction of bicyclo[1.1.0]butanes (BCBs) **32** with α-alkenylazides **33**, achieving divergent synthesis of 2- and 3-azabicyclo[3.1.1]heptenes (aza-BCHepes) **35** or **36** (Scheme 9) [32]. This study developed a practical method for constructing novel 2- and 3-aza[3.1.1]heptene architectures from readily available α-alkenylazides and BCBs through catalyst-controlled (3 + 3) and (3 + 2) cyclization strategies. Two distinct pathways were established: (1) The titanium-catalyzed ring opening of bicyclobutane (BCB) **32** generates a γ-carbonyl radical intermediate **Int-42**, which undergoes trapping by vinylazide **33**. Subsequent dinitrogen extrusion produces an iminyl radical species **Int-44**. This reactive intermediate then engages with a Ti(IV)-enolate complex through radical recombination, ultimately delivering 2-aza-bicyclo[3.1.1]heptene (BCHepe) while regenerating the Ti(III) catalyst to complete the catalytic cycle. (2) Scandium-catalyzed pathway: Activation of the donor–acceptor BCB via Sc(OTf)_3_ coordination to its carbonyl group facilitates nucleophilic attack by vinylazide **33**, forming an imino-diazonium intermediate **Int-40** accompanied by a δ-carbanion. Transannular cyclization of this species affords 2-azidobicyclo[3.1.1]hexane (2-azidoBCHs). Subsequent thermal activation induces selective migration of the less sterically hindered secondary carbon center with concomitant dinitrogen elimination, yielding 3-aza-BCHepe as the final product.

### Solvent control

**Scheme 10:**
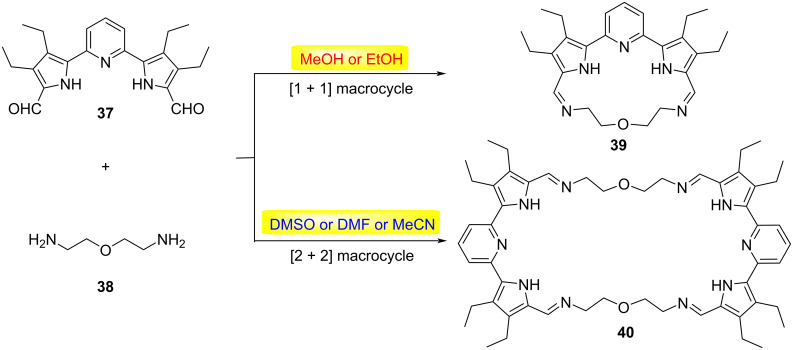
Solvent-driven reversible macrocycle-to-macrocycle interconversion [39].

The solvent microenvironment emerged as a critical determinant in governing stereochemical outcomes, exerting profound influence through multifaceted solute–solvent interactions [5]. Solvent polarity, hydrogen-bonding propensity, and dielectric characteristics collectively orchestrate stereodivergent pathways through dynamic coordination effects and differential stabilization of transition states. Notably, these solvent-mediated electronic and steric modulations frequently dictate reaction stereoselectivity, with even subtle solvent permutations potentially inducing complete stereochemical inversion in sensitive systems [33–38]. In 2023, He and Sessler disclosed a versatile one-pot synthesis of structurally diverse macrocycles through the dynamic self-assembly of α,α’-linked oligopyrrolic dialdehydes and alkyldiamines (Scheme 10) [39]. Their investigation revealed distinct solvent-mediated selectivity in product formation. Condensation of the pyridine-bridged oligopyrrolic dialdehyde **37** with simple alkyldiamines proceeded with solvent-independent regioselectivity, exclusively furnished [2 + 2] macrocyclic adducts. Strikingly, when **37** was combined with 2,2’-oxybis(ethylamine) (**38**), the reaction pathway exhibited pronounced solvent dependency. Reactions in methanol, ethanol, or chloroform selectively generated the [1 + 1] macrocycle **39** as the sole product. In contrast, polar aprotic solvents such as dimethyl sulfoxide (DMSO), *N*,*N*-dimethylformamide (DMF), or acetonitrile (MeCN) favored precipitation of the [2 + 2] macrocycle **40**. Notably, the macrocycle **40** underwent spontaneous structural reorganization in chloroform or dichloromethane (DCM), converting entirely into the thermodynamically stable [1 + 1] isomer **39**. This work demonstrates a solvent-driven approach for dynamically interconverting macrocycle sizes, governed by thermodynamic stability and solubility differences.

**Scheme 11:**
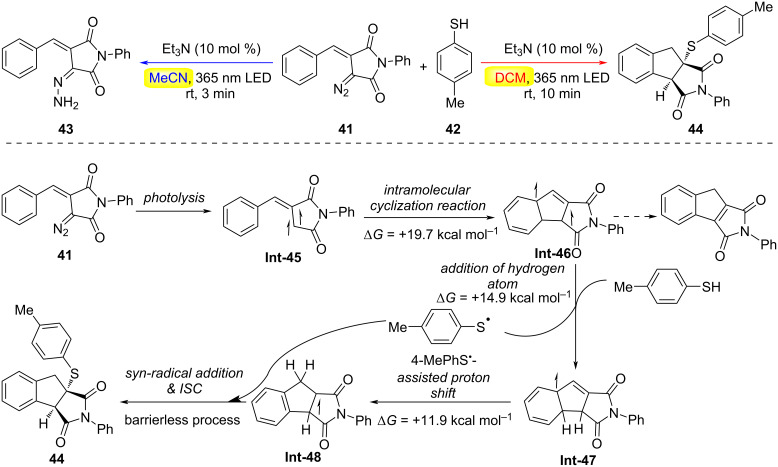
Unexpected solvent-dependent reactivity of cyclic diazo imides and mechanism [40].

In the same year, Chauhan, Koenigs, and co-workers demonstrated solvent-controlled bifurcation in the light-driven reactivity of cyclic diazo imides **41** with thiols **42**, unveiling two mechanistically distinct pathways (Scheme 11) [40]. In dichloromethane (DCM), the reaction proceeds via a carbene intermediate, enabling cascade C(sp^2^)–H functionalization/thiolation to deliver indane-fused pyrrolidines **43** in excellent yields (up to 92%). Strikingly, switching the solvent to acetonitrile completely suppresses carbene formation under identical conditions, redirecting the pathway toward an unconventional diazo reduction wherein aryl thiols act as stoichiometric reductants. Mechanistic insights, elucidated through control experiments and DFT calculations, revealed that photoexcitation of diazo imide **41** triggers nitrogen extrusion (Δ*G*^‡^ = +10.0 kcal·mol^−1^), generating the triplet carbene intermediate **Int-45**. In DCM, this species undergoes intramolecular cyclization into a proximal C(sp^2^)–H bond (Δ*G*^‡^ = +19.7 kcal·mol^−1^) to form **Int-46**, which reacts with 4-MePhSH (Δ*G*^‡^ = +14.9 kcal·mol^−1^) to yield radical intermediate **Int-47** and a thiyl radical (4-MePhS**^·^**). Sequential thiol-assisted hydrogen shifts produce **Int-48**, followed by barrierless thiyl radical addition and intersystem crossing (ISC) to furnish the final product. In contrast, acetonitrile’s polar aprotic environment destabilizes the carbene pathway, favoring direct reduction of the diazo moiety via electron transfer from the thiol. This solvent-gated selectivity underscores the critical role of reaction medium polarity in modulating reactive intermediates, offering a strategic lever to toggle between C–H functionalization and reductive manifolds in photochemical transformations.

**Scheme 12:**
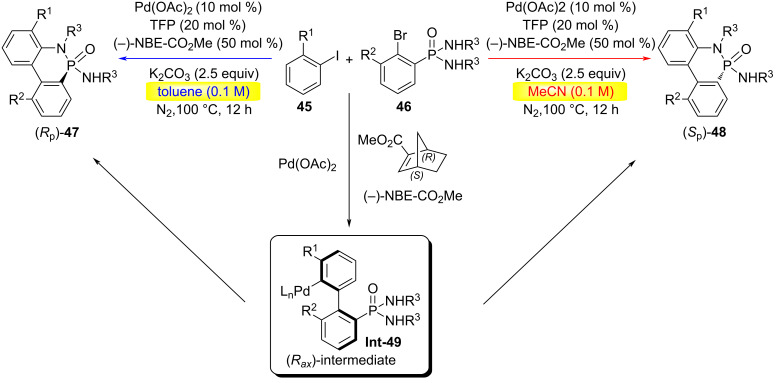
Palladium-catalyzed annulation of prochiral *N*-arylphosphonamides with aromatic iodides [41].

In 2024, the Cheng group developed a palladium/chiral norbornene (NBE)-catalyzed cyclization reaction between aryl iodides **45** and phosphoramides **46** under varying solvent conditions of toluene (PhMe) and acetonitrile (MeCN), based on their studies of the Catellani reaction (Scheme 12) [41]. This method exhibited a broad substrate scope for both aryl iodides and phosphoramides, and enabled enantioselective access to both enantiomers of chiral P(V) molecules **47** or **48** using a single chiral NBE catalyst.

### Time control

**Scheme 13:**
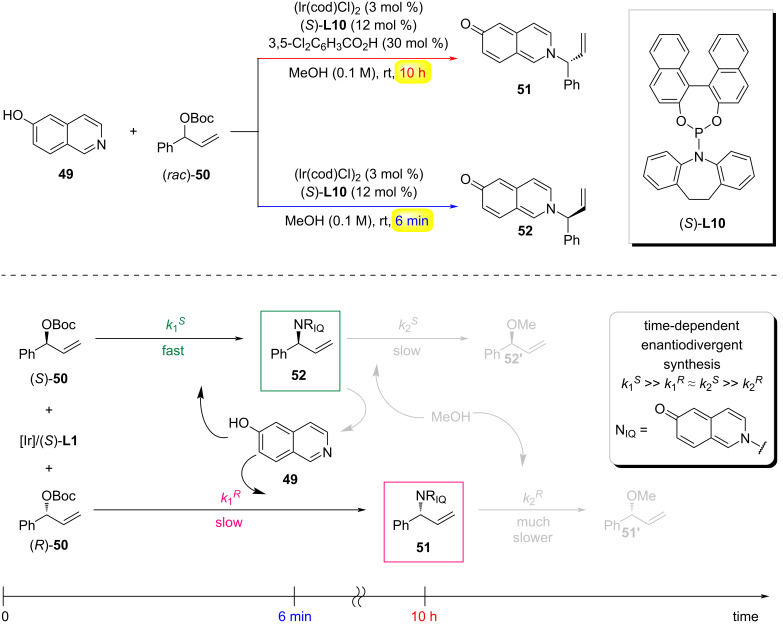
Time-dependent enantiodivergent synthesis [42].

Time control of chemical reactivity offers an inherent strategy to program synthetic pathways through kinetic discrimination of transient intermediates. Diverging from additive-dependent or stimulus-responsive approaches, this paradigm capitalizes on the chronoselective evolution of reactive species to unlock sequence-controlled transformations. In 2020, the You group reported a reaction-time-dependent enantiodivergent synthesis method. Under the same chiral catalytic system, they achieved selective synthesis of either enantiomer of a target product by controlling the reaction duration (Scheme 13) [42]. When performing the asymmetric intermolecular allylic amination of 6-hydroxyisoquinoline (**49**) with *tert*-butyl(1-phenylallyl)carbonate ((*rac*)-**50**) using an Ir catalyst derived from [Ir(cod)Cl]_2_ and the Carreira chiral phosphoramidite ligand (*S*)-**L10**, along with the addition of 3,5-dichlorobenzoic acid as an additive in MeOH at room temperature, the reaction proceeded smoothly for 10 hours to yield the aminated product **51**. Interestingly, when the reaction was quenched after 6 minutes in the absence of a Brønsted acid additive, the opposite enantiomer **52** was obtained. Mechanistically, an initial kinetic resolution (KR) of (*rac*)-**50** occurs via an Ir-catalyzed asymmetric allylic amination. Due to the higher reactivity of (*S*)-**50**, it reacts with 6-hydroxyisoquinoline within 6 minutes to generate **52**, while (*R*)-**50** remains largely unreacted during this period (*k*_1_*^R ^<< k*_1_*^S^*). However, as the reaction progresses, **52** undergoes further reaction with MeOH under the catalytic system to form **52'**. Meanwhile, the less reactive (*R*)-**50** gradually reacts with 6-hydroxyisoquinoline, leading to the accumulation of **51**. Since **51** is highly stable and resistant to reaction with MeOH (*k*_2_*^R^* << *k*_2_*^S^* ≈ *k*_1_*^R^*), it can be obtained with high optical purity after an extended reaction time (10 hours)

**Scheme 14:**
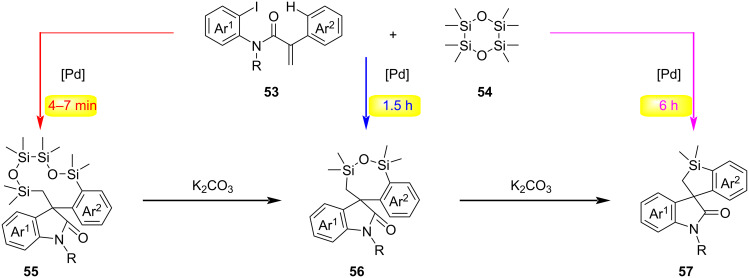
Time-controlled palladium-catalyzed divergent synthesis of silacycles via C–H activation [43].

**Scheme 15:**
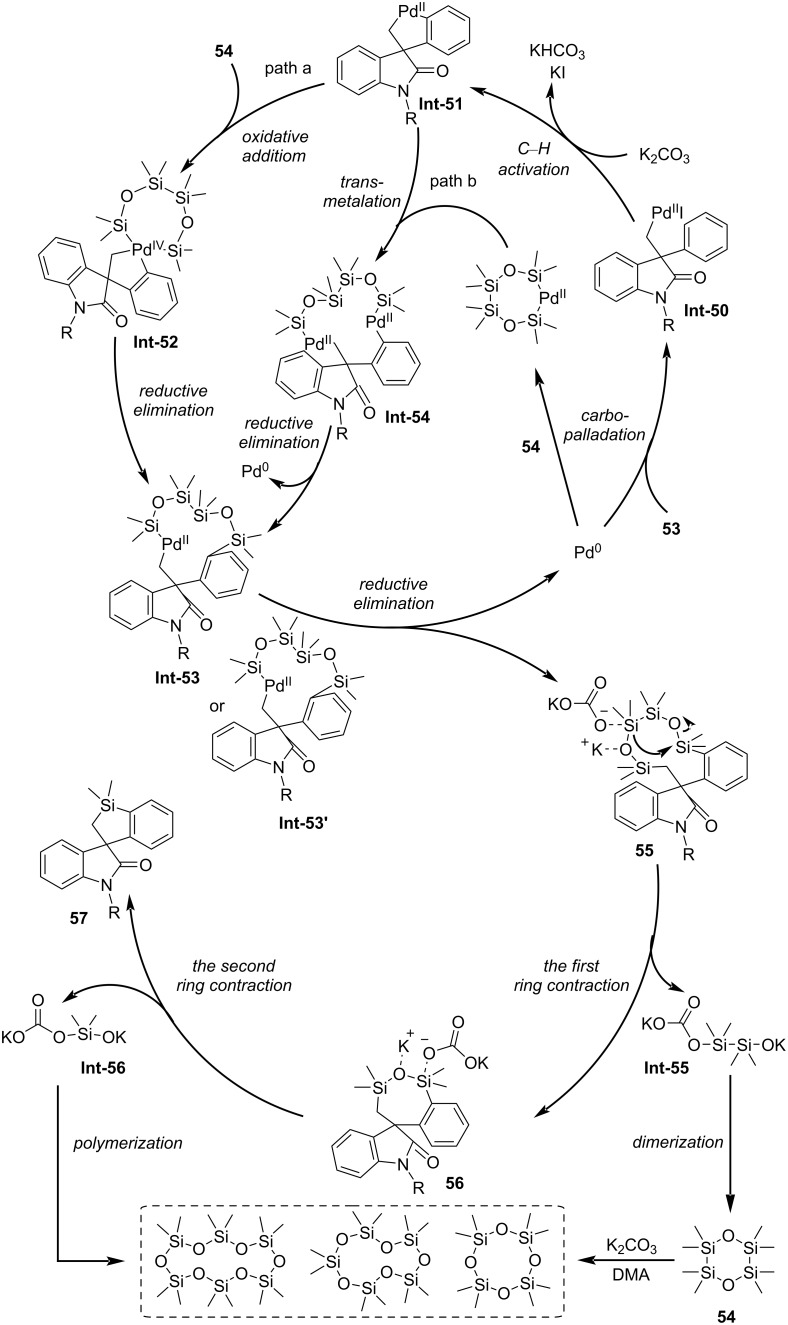
Proposed mechanism for the time-controlled palladium-catalyzed divergent synthesis of silacycles [43].

In 2023, Yang and Liang jointly reported a tetrasilane (ODCS)-based method for time-controlled, palladium-catalyzed C–H activation in the divergent synthesis of silacyclic compounds (Scheme 14) [43]. This reaction employs the ODCS reagent to capture a five-membered C,C-palladacycle species, using reaction time as a control switch to enable transformations of three distinct substrates – acrylamides, 2-halo-*N*-methylacryloylbenzamides, and 2-iodobiphenyls – thereby selectively synthesizing silacyclic compounds with varying ring sizes, including ten-membered, seven-membered, and five-membered rings. Mechanism (Scheme 15): Substrate **53** undergoes oxidative addition with Pd(0), followed by intramolecular carbopalladation to form the σ-alkylpalladium intermediate **Int-50**. The intermediate **Int-50** undergoes C–H activation to generate the spiro-palladacycle **Int-51**, which proceeds via two possible pathways: 1) Path a: oxidative addition/reductive elimination or 2) path b: transmetalation/reductive elimination giving rise to intermediates **Int-53** or **Int-53'**. Reductive elimination of **Int-53** or **Int-53'** regenerates Pd(0) and produces intermediate **55**. With the assistance of the base K_2_CO_3_, the ten-membered silacycle **55** undergoes rapid ring contraction via cleavage of two Si–O bonds and formation of one Si–O bond, leading to **56** and **Int-55**. Concurrently, **Int-55** dimerizes to form **54**, which is further transformed into cyclosiloxanes under K_2_CO_3_ and DMA conditions. Intermediate **56** undergoes additional ring contraction through cleavage of Si–O/Si–C bonds and formation of a Si–C bond, yielding **57** and **Int-56**, with **Int-56** polymerizing to generate cyclosiloxanes. An alternative pathway involving cleavage of another Si–O bond during the conversion from **55** to **56** and subsequently to **57** cannot be excluded.

### Temperature control

**Scheme 16:**
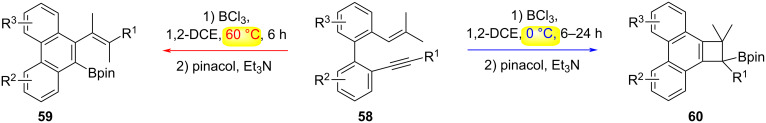
Metal-free temperature-controlled regiodivergent borylative cyclizations of enynes [45].

Temperature, as a readily adjustable physical parameter in organic synthesis, offers a simple and versatile approach to control regioselectivity. It profoundly influences reaction kinetics, stability of intermediates, and reaction equilibria. Through precise temperature modulation, chemists can effectively steer the formation of regioisomers, often achieving desired selectivity with minimal alterations to other reaction components. For instance, Rana and colleagues developed a temperature-dependent regiodivergent strategy to access functionalized maleimides and itaconimides [44]. This thermochemical strategy provides a robust platform for controlling reaction pathways while maintaining synthetic simplicity. In 2022, García-García and Fernández-Rodríguez reported on the practicality of metal-free BCl_3_-catalyzed borylation cyclization reactions in synthesis (Scheme 16) [45]. Biphenyl-embedded 1,3,5-trienes-7-yne compounds **58** react with BCl_3_ under catalyst-free and additive-free conditions to form novel polycyclic boronated structural units. By adjusting the temperature of the reaction medium, it is possible to precisely control the reaction pathway, thereby obtaining two different boronated frameworks from the same starting material: boronated phenanthrene derivatives **59** at 60 °C and phenanthrene-fused boronated cyclobutane **60** at 0 °C.

**Scheme 17:**
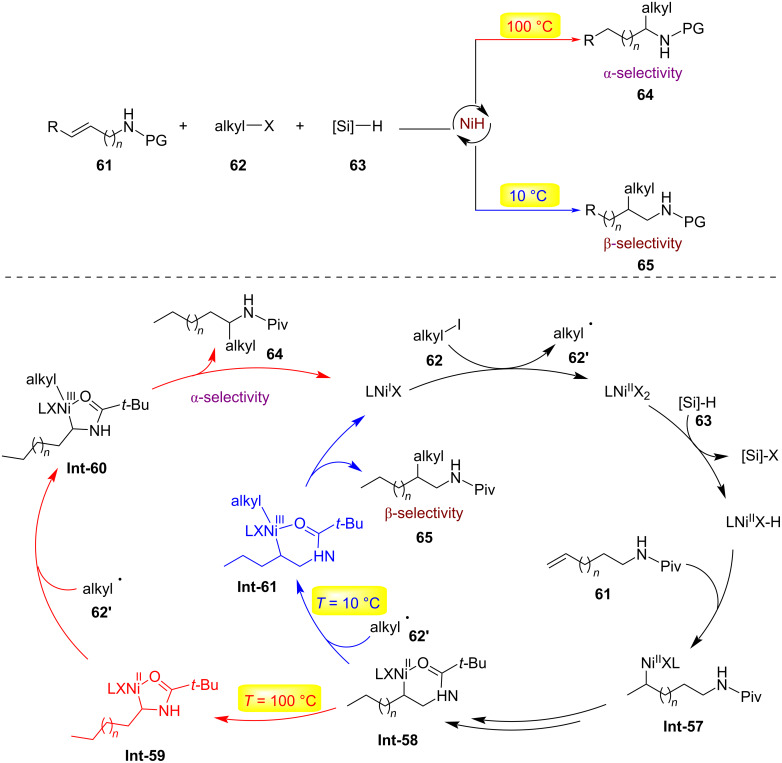
Nickel-catalyzed switchable site-selective alkene hydroalkylation by temperature regulation [46].

In the same year, Lu's research group reported a temperature-controlled site-selective olefin hydroalkylation reaction (Scheme 17) [46]. By adjusting only the reaction temperature, different skeletal structures of nitrogen α- and β-alkylated products could be obtained from the same olefin substrates **61**. At 10 °C, the catalytic system consisting of NiBr₂(diglyme), oxazoline ligand, (EtO)₃SiH, and K₃PO₄(H₂O) achieved β-selective hydroalkylation. When the temperature was raised to 100 °C, the reaction selectively produced α-branched products. DFT calculations showed that at low temperatures, the six-membered nickel ring captures radicals and undergoes reductive elimination to form β-products (kinetic control); at high temperatures, the formation of a five-membered nickel ring leads to α-products (thermodynamic control). Therefore, the formation of the more stable nickel ring drives migration, while the thermodynamic and kinetic properties of different reductive elimination intermediates jointly determine the switchable site selectivity.

### Acid–base control

**Scheme 18:**
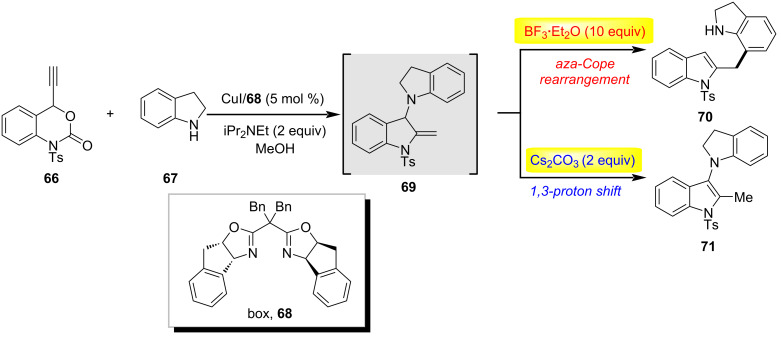
Copper-catalyzed decarboxylative amination/hydroamination sequence [48].

**Scheme 19:**
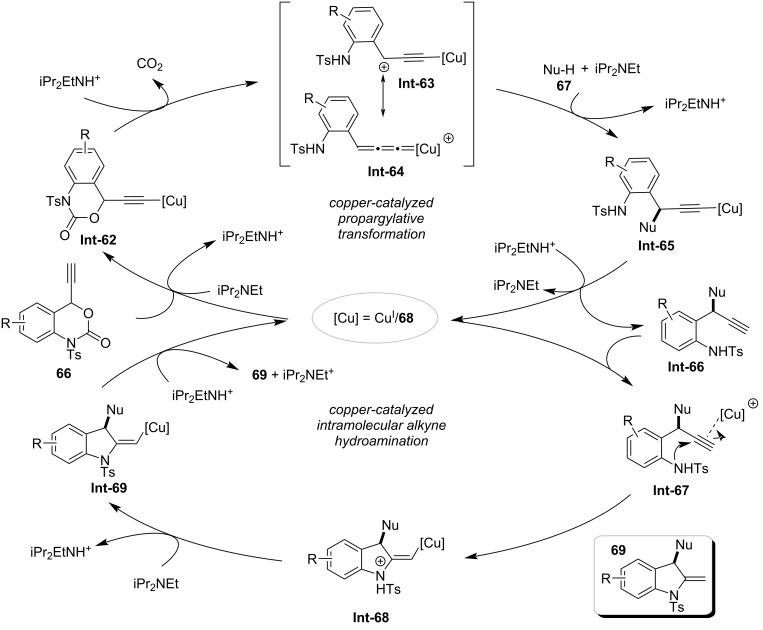
Proposed mechanism of copper-catalyzed decarboxylative amination/hydroamination sequence [48].

The strategic modulation of acid–base interactions has emerged as a powerful paradigm in organic synthesis, enabling precise control over reaction pathways, selectivity, and catalytic efficiency. By exploiting dynamic acid–base equilibria or stimuli-responsive systems, chemists can manipulate substrate activation, stabilize reactive intermediates, and orchestrate complex multistep transformations under mild conditions [47]. In 2016, Lu's team designed a new class of acetylene carbonate reagents and successfully applied them to copper-catalyzed decarboxylative amination/hydroamination sequences (Scheme 18) [48]. By controlling acidic and basic reaction conditions, the authors achieved the controllable synthesis of two types of functionalized indoles. When treated with acid (BF_3_·E_2_O), the intermediate 2-methylene-3-aminoindoline **69** undergoes an aza-Cope rearrangement to form 2-benzylindole **70**; when treated with a base (Cs_2_CO_3_), this intermediate undergoes a 1,3-proton migration process to convert back to 3-aminoindole **71**. The possible mechanism for the formation of the key intermediate **69** is outlined in Scheme 19: first, substrate **67**, under the action of a copper catalyst and diisopropylethylamine, undergoes a decarboxylation process to generate the allylidenecopper intermediate **Int-63** and its resonance form **Int-64**. Subsequently, these intermediates undergo a propargylation process (**Int-63**, **Int-64** to **Int-65**) followed by a proton elimination process to generate **Int-66** (**Int-5** to **Int-66**). Then, **Int-66** undergoes an intramolecular amination through copper-catalyzed activation to form **Int-68**, and finally, 2-methylene-3-aminoindoline **69** is generated via a proton transfer promoted by diisopropylethylamine.

**Scheme 20:**
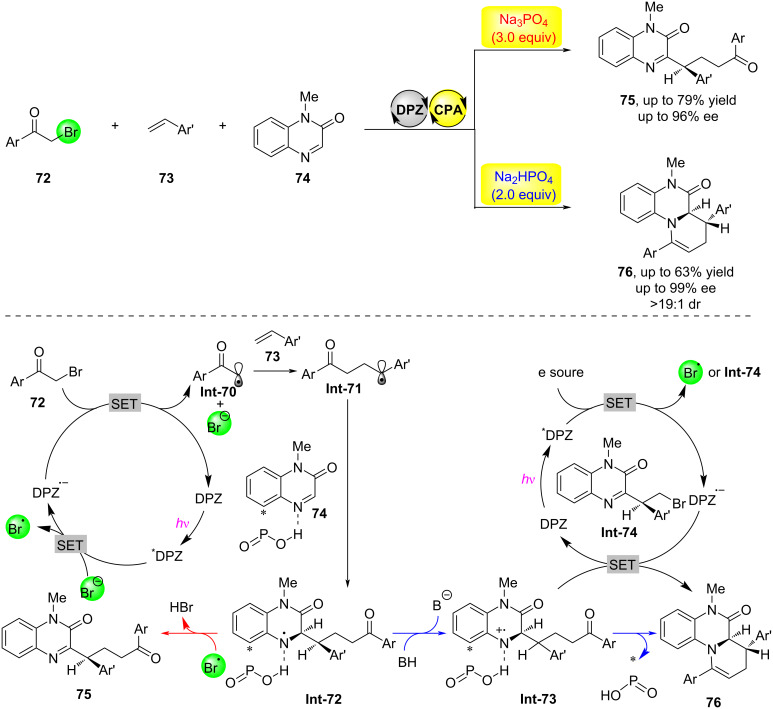
Enantioselective chemodivergent three-component radical tandem reactions [49].

In 2023, the Jiang research group achieved a chemically divergent photocatalytic asymmetric synthesis using a dual catalytic system consisting of a chiral phosphoric acid and dicyanopyrazine (DPZ) as the photosensitizer (Scheme 20) [49]. By regulating the chemical selectivity of a three-component radical cascade reaction involving α-brominated aryl ketones **72**, olefins **73**, and 1-methylquinoxalin-2(1*H*)-one (**74**) with an inorganic base, they were able to obtain two important types of products with high yield and enantioselectivity. Through mechanistic experiments and DFT calculations, the authors proposed a possible mechanism for the reaction: first, DPZ is excited by light to form the excited state DPZ*, which then oxidizes bromide ions through single-electron transfer to generate corresponding radical anions. These radical anions undergo single-electron transfer with substrate **72** to form radical intermediate **Int-70**, completing the DPZ catalytic cycle. Intermediate **Int-70** adds to substrate **73** to form radical intermediate **Int-71**, which further adds to hydrogen-bond-activated substrate **74** to form hydrogen-bonded complex **Int-72**. When Na_3_PO_4_ is used as the inorganic base, bromine radicals abstract hydrogen to form product **75**; whereas when Na_2_HPO_4_ is used as the inorganic base, its weaker basicity leads to protonation of complex **Int-72** to form intermediate **Int-73**, which then preferentially undergoes single-electron transfer with the DPZ radical anion, followed by cyclization and dehydration to yield bicyclic product **76**.

### Substrate control

**Scheme 21:**
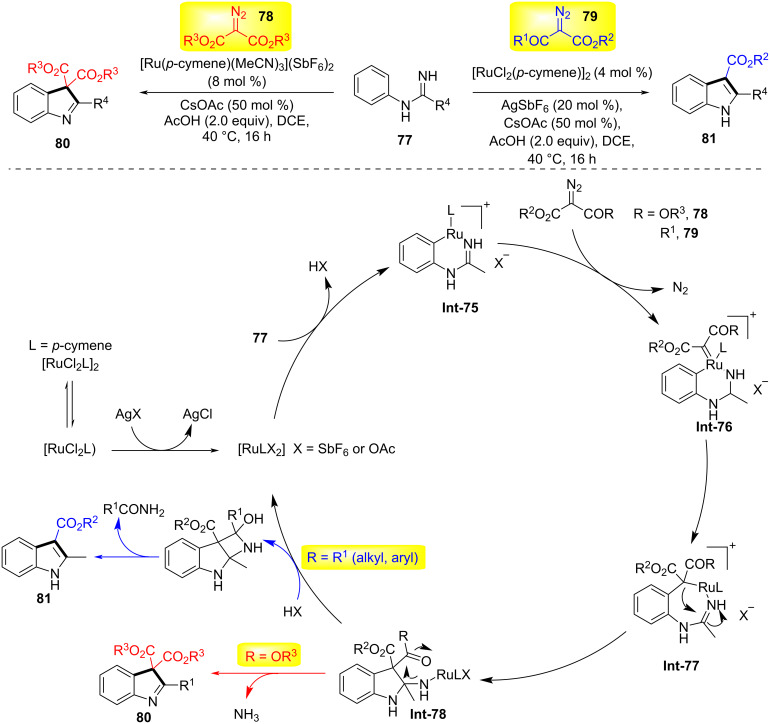
Substrate-controlled synthesis of indoles and 3*H*-indoles [52].

Substrate control has emerged as a powerful strategy in organic synthesis, enabling precise manipulation of reaction pathways and stereochemical outcomes through the intrinsic structural and electronic features of the starting material. By exploiting preorganization, steric effects, or directing groups within the substrate, chemists can achieve high levels of regioselectivity, diastereoselectivity, and enantioselectivity without relying on external catalysts or additives. This approach has been successfully applied in the synthesis of complex natural products, pharmaceuticals, and functional materials, often streamlining multistep sequences and minimizing protecting-group strategies [50–51]. In 2016, Li and co-workers developed divergent coupling conditions for iminamides **77** with receptor-type diazo compounds **78** or **79** under ruthenium catalysis, generating indoles **81** and 3*H*-indoles **80**, respectively (Scheme 21) [52]. α-Diazo-β-ketoesters form indoles by cleaving the C(N₂)–C(acyl) bond, while diazomalonates form 3*H*-indoles through C–N-bond cleavage. Mechanistically, the cyclometalation of iminamides follows a concerted metalation–deprotonation (CMD) mechanism to generate ruthenium intermediate **Int-75**. Subsequently, diazo compound **78** or **79** coordinates with intermediate **Int-75**, followed by deazidation to form the ruthenium carbenoid species **Int-76**. The ruthenium–aryl bond in this intermediate migrates into the carbenoid unit, providing heptacyclic ruthenium ring intermediate **Int-77**. Intermediate **Int-78** is then formed via ruthenium migration insertion into the C=N bond from Ru–C(alkyl). For diazoketoester substrates, the final product **81** is released from **Int-78** through protonation, intramolecular nucleophilic addition, and subsequent release of one molecule of amide, reactivating the active ruthenium(II) catalyst. In contrast, for diazomalonates, intermediate **Int-78** releases ammonia with the help of Ru(II) or acetic acid, ultimately yielding 3*H*-indole **80**. This change in selectivity may be due to the reduced electrophilicity of the ester carbonyl.

**Scheme 22:**
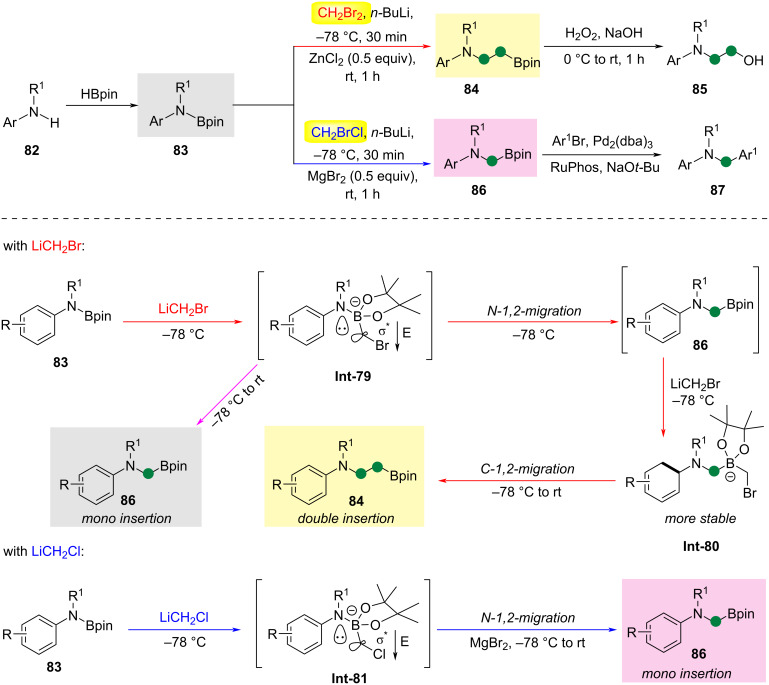
Controlled mono- and double methylene insertions into nitrogen–boron bonds [53].

In 2021, Dong and Xie reported the development of an azido Matteson reaction, which achieves carbene insertion into an N–B bond of aminoboranes **84** or **86** (Scheme 22) [53]. In this methodology, by controlling the carbene leaving group (alkyl chlorides/alkyl bromides) and the Lewis acid activator, a selective mono- or di-methylene insertion reaction can be carried out, generating α-/β-boryl-substituted tertiary organic amines **83** from simple secondary organic amines. Using *N*-alkyl-*N*-arylaminoboranes as the reactant, the reaction proceeds at −78 °C with CH_2_Br_2_ and *n*-BuLi, followed by a reaction with ZnCl_2_ at room temperature. The product is then hydrolyzed with a NaOH solution of H_2_O_2_ to yield amino alcohols. The mechanism involves the formation of borate intermediate **Int-79** from substrate **83** under the action of CH_2_BrLi. This is followed by an N-1,2-migration to form borate ester **86**, which then reacts with another molecule of CH_2_BrLi to form the more stable borate **Int-80**. Subsequently, a C-1,2-migration leads to the formation of the double-insertion product **84**. If the amine portion is more electron-deficient or has more delocalized nitrogen electrons (such as indole substrates), **Int-79** is more stable at −78 °C, favoring the formation of the mono-insertion product **86**.

**Scheme 23:**
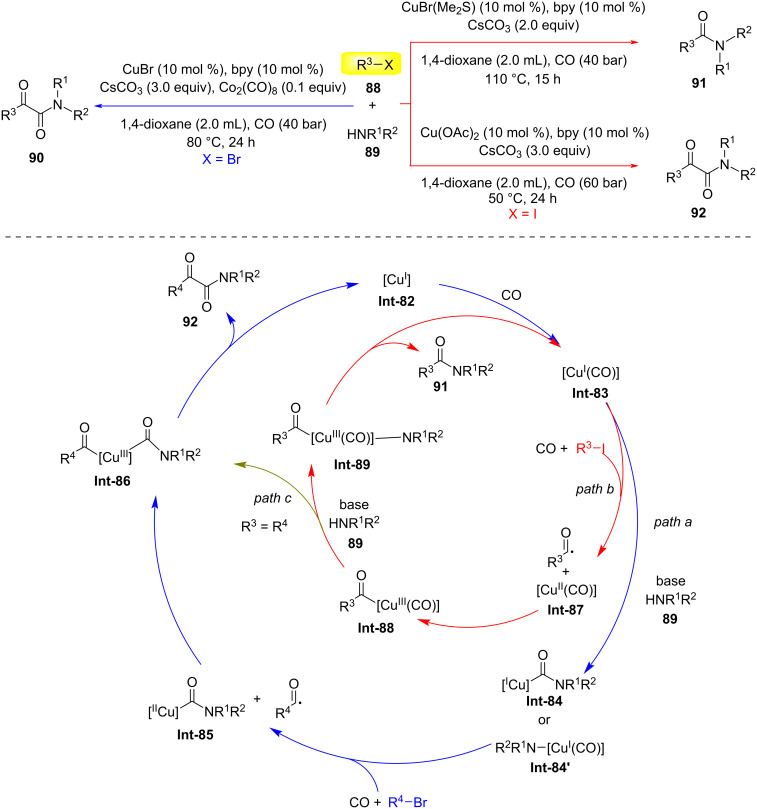
Copper-catalyzed substrate-controlled carbonylative synthesis of α-keto amides and amides [54].

In 2022, Wu and colleagues reported a novel methodology for constructing α-ketoamides **90** or **92** and amides **91** through copper-catalyzed dicarbonylation and monocarbonylation reactions involving alkyl halides **88** (Scheme 23) [54]. Using alkyl bromides, CuBr as the catalyst, bpy as the ligand, Co_2_(CO)_8_ as the additive, Cs_2_CO_3_ as the base, and 1,4-dioxane as the solvent under 40 bar CO pressure at 80 °C, they successfully synthesized α-ketoamides **90**. When alkyl iodides were used as substrates, both dicarbonylation and monocarbonylation processes occurred simultaneously with Cu(OAc)_2_, favoring the dicarbonylation process. In contrast, using CuBr(Me_2_S) the monocarbonylation process was favored. Possible reaction mechanisms: First, CO coordinates with copper salts to form (carbonyl)copper species **Int-83**. Subsequently, in the presence of a base, the amine undergoes nucleophilic attack on the coordinated CO, generating (carbamoyl)copper complex **Int-84**. Then, alkyl bromide undergoes a single-electron-transfer (SET) process with **Int-84**, forming intermediate **Int-85** and an alkyl radical, which is captured by CO to yield an acyl radical. Alternatively, under the action of a base, the amine can undergo anionic ligand exchange with (carbonyl)copper species **Int-83**, generating an electron-rich amino copper(I) species **Int-84'**, which activates alkyl bromide through an SET process, followed by immediate insertion of CO to form complex **Int-85**. Nucleophilic activation of the acyl radical initiates through its reaction with intermediate **Int-85**, generating the critical acyl(aminoacyl)copper species **Int-86**. Subsequent reductive elimination from this intermediate liberates the α-ketoamide product **92** while regenerating the catalytic species **Int-82**. Comparative kinetic analysis revealed a marked preference for alkyl iodide activation, as demonstrated by its substantially lower activation energy barrier compared to alkyl bromide analogs (path b). This energetic advantage facilitates preferential formation of intermediate **Int-87** via oxidative addition. Rapid coupling with the in situ-generated acyl radical produces copper-bound intermediate **Int-88**. Base-mediated anionic exchange then displaces the halide ligand with amine, yielding intermediate **Int-89**. Final reductive elimination from this species affords amide product **91** with concurrent regeneration of the catalyst **Int-83**. Notably, a competitive pathway emerges through alternative reactivity of **Int-88** (path c). The coordinated CO ligand undergoes nucleophilic attack by the amine, bypassing halide exchange to instead generate **Int-86**. This mechanistic crossover establishes a product dichotomy between α-ketoamide **92** and amide **91**, with the branching ratio governed by relative rates of base-mediated exchange versus CO activation at the copper center.

**Scheme 24:**
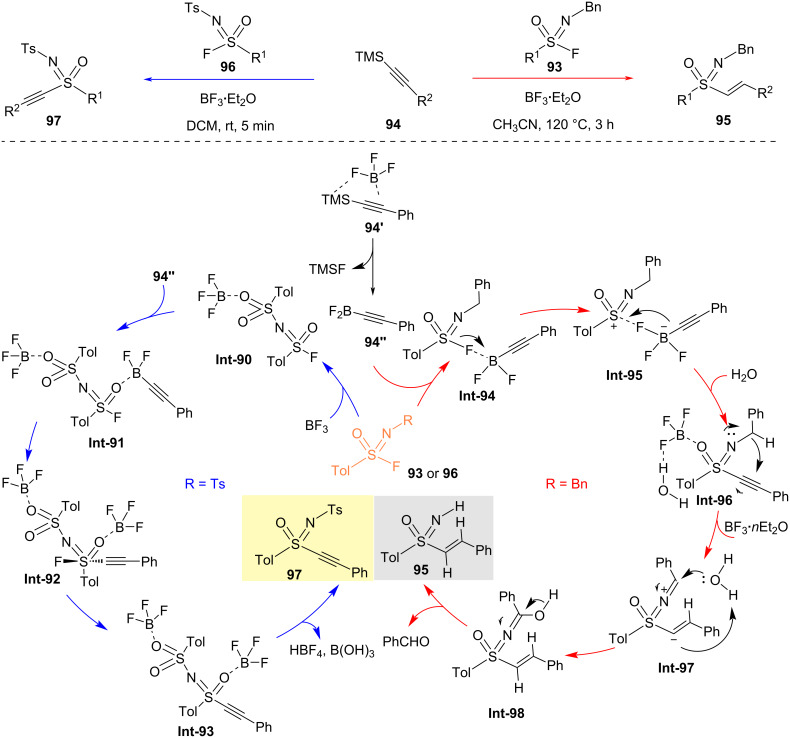
Divergent sulfur(VI) fluoride exchange linkage of sulfonimidoyl fluorides and alkynes [55].

In 2022, the Jiang research team developed regulated SuFEx click chemistry between fluorosulfonyl imides and TMS-alkynes, enabling the rapid construction of S(VI)–C(sp^2^) or S(VI)–C(sp) bonds efficiently (Scheme 24) [55]. This linkage utilizes the high bond dissociation energy (BDE = 135 kcal/mol) of silicon–fluorine bonds, employing trifluoroborate as a fluorine transfer reagent to simultaneously cleave the S(VI)–F bond and activate the Si–C bond. DFT calculations indicate that the reaction proceeds via the formation of a difluoroborate phenylacetylene intermediate **94’’** by in situ generation from boron trifluoride etherate and silicon-protected phenylacetylene **94**, which activates the S–F bond of the fluorosulfonyl imide to form sulfonyliminium cations **Int-95**. These then add to the activated phenylacetylene to construct the S–C bond, followed by intramolecular 1,5-hydrogen migration and aqueous workup to remove benzaldehyde, yielding the target sulfonylimine products.

**Scheme 25:**
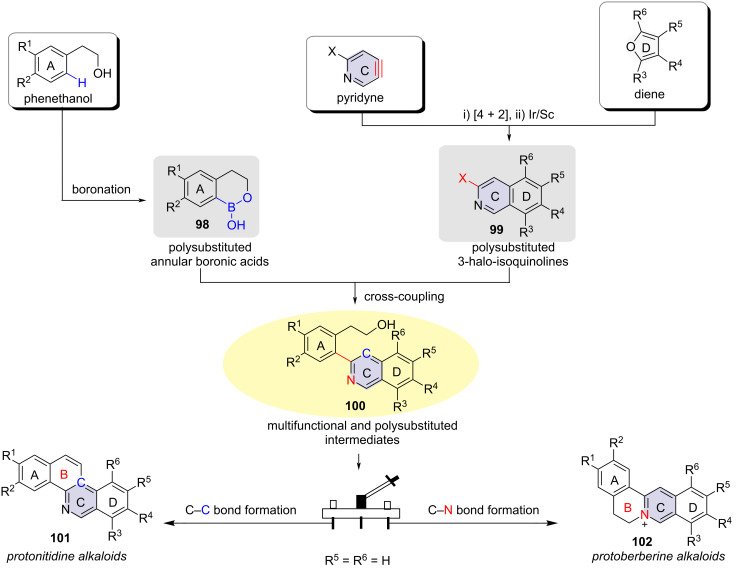
Modular and divergent syntheses of protoberberine and protonitidine alkaloids [56].

Both original protoberberine and protonitidine alkaloids are characterized by an isoquinoline ring skeleton. An analysis of their molecular structures revealed that the two alkaloids share a basic structure, differing only in the junction of the B-ring. In 2021, Liu and Jiang designed new pyridyne precursors, which underwent cycloaddition reactions with substituted furans as diene component to produce the corresponding epoxy-cycloaddition adducts. The authors developed an Ir/Sc tandem catalytic reaction to convert these adducts into polysubstituted 3-haloisoquinolines **99** in one pot. After obtaining isoquinoline compounds **99** with different substituents and polysubstituted annular boronic acids **98**, a Suzuki coupling was employed to synthesize advanced isoquinoline intermediates **100**. Following this, a 6π electrocyclization reaction and nucleophilic reaction were developed to achieve C–C and C–N bond constructions, respectively, leading to the synthesis of differently substituted protonitidine alkaloids and protoberberine alkaloids (Scheme 25) [56].

## Conclusion

Developing streamlined and versatile approaches for the rapid assembly of structurally diverse organic molecules represents a pivotal challenge in organic synthesis, pharmaceutical research, and advanced materials development. Recent advances in controllable/divergent synthesis methodologies, which enable the construction of variously functionalized architectures from common precursors, have emerged as particularly promising due to their inherent efficiency. Contemporary strategies for controlling reaction pathways and selectivity predominantly involve precise manipulation of catalytic systems (metal catalysts/ligands), reaction parameters (solvent, temperature, time), acid/base mediation, and strategic substrate engineering. This review systematically organizes recent breakthroughs according to critical control elements governing product divergence. Through mechanistic investigations of pivotal bond-forming steps and comparative analysis of representative case studies, we provide fundamental insights into the origin of selectivity variations and reaction pathway control. The discussion emphasizes structure–reactivity relationships and catalytic design principles that enable predictable access to distinct molecular architectures from shared synthetic intermediates. This review serves as a conceptualized platform for controllable/divergent synthesis, arousing more state-of-the-art tactics in chemical synthesis.

## Data Availability

Data sharing is not applicable as no new data was generated or analyzed in this study.
